# Book Reviews

**Published:** 1825-02

**Authors:** 


### CRITICAL ANALYSIS
OF
### ENGLISH AND FOREIGN LITERATURE
RELATIVE TO THE VARIOUS BRANCHES OF
### Medical Science.

Quæ laudanda forent, et quæ culpanda, vicissim

Illa, prius, cretâ; mox hæc, carbone, *notamus*.—PERSIUS.

### DIVISION I.

### ENGLISH.

ART. I.—*Clinical Report on Dropsies; with Observations explanatory of their Pathology and Therapeutics. With an Appendix, on the Theory and Treatment of Organic Disease in general.* By ROBERT VENABLES, Bachelor in Medicine, and Licentiate in Physic of the University of Oxford; Physician to the Henley Dispensary, and Consulting Physician to the Poor-house.—London: T. and G. Underwood, 1824. 8vo. pp. 238.

DR. VENABLES, though a bachelor in medicine, is by no means a tyro in the practice of his profession. We are informed, in the Preface, that he has enjoyed fifteen years' observation of diseases in different climates, and under almost every variety of circumstances; and, from what is stated at page 159, we may gather that he has been attached, during some portion of that period, to the medical department of the Ordnance. We looked forward, therefore, with much eagerness to the results of his observation and experience; and (as is our custom in these cases) we first directed our attention to discover the principal jet and aim of the author. Our eye was instantly attracted by the delightful announcement, in the very first page of the Preface, that "the view here taken of the theory of dropsy is in some degree novel." Entering freely into our author's feelings regarding the importance of the investigation, we indulged for a few moments in pleasing anticipations. We imagined to ourselves the subject of dropsical effusions divested of some, at least, of those difficulties with which we had long felt it to be involved, and looked forward with real pleasure to the additional means of combating this formidable enemy, which we fully expected would result from the *new* views of our author. We confess ourselves to have been rather disappointed, therefore, in finding, after a careful perusal of the volume, that the principal objects of the author were (in theory) to demonstrate that dropsy is frequently a functional disease, and that it has not that necessary connexion with organic derangement which was once supposed; and (in practice) that dropsies were often curable without the aid of diuretics. The author indulges, indeed, as we shall presently see, in some other speculations, theoretical and practical; but the spirit and scope of the author's reasonings, as far as dropsy is concerned, is to prove the truth of the two propositions above mentioned.

Now here, we think, we can see very clearly one of the disadvantages of country practice. Had this gentleman been in town, attending occasionally at any of our great hospitals, and conversing with the medical men, both young and old, whom he would there meet, he would never have fallen into such an error as to imagine that it was necessary, in the year 1824, to publish a volume of 238 pages to prove what, we believe, is generally acknowledged. They were among the first lessons we were ever taught in the theory of dropsy; nor did we for a moment dream that we were in possession of any secret, till Dr. Venables' book fell into our hands. It is possible, however, that we may be mistaken;—there may be practitioners and pupils who are ignorant of the great pathological principle, that dropsy is, in very many cases, a functional disorder. For them the work will undoubtedly have its attractions; and we can confidently assure all such, that the facts and reasonings of the author will convince the most sceptical of the firm foundation on which the opinion rests.

The *great* objection, then, (as our readers will see,) which we have to the book, is, not that it is incorrect, but that it is uncalled for. All that is valuable in it has been acknowledged as such as long as our memory serves us, and doubtless long before our era. But we have other errors to notice, independent of this great and radical one. We observe in the author, in the first place, a disposition to find fault with the practice of his professional brethren, which is bad enough in common conversation, but is really very censurable in print. We will not add to the mischief by quoting the anecdote at page ix. of the Preface; but we would recommend to the author to expunge this obnoxious passage in that larger work on Dropsy, which he tells us he is now preparing, with the assistance of the clumsy work of Portal. Dr. Venables should recollect that we are all liable to error, and that indulgently to overlook the faults of our professional brethren, is only a branch of that golden rule which teaches us to do unto others as we would that they should do unto us.

Our next cause of complaint with Dr. Venables, is an occasional unfairness in his way of stating cases. We have a striking example of this at page 68, in his observations on one of the cases which he brings forward. It was from the first a very doubtful, if not nearly hopeless, one. A young man had a deep, eroding, angry-looking, syphilitic ulcer upon the glans penis: his constitution was highly irritable; mercury aggravated the inflammatory excitement; symptoms of peritoneal inflammation and dropsy first appeared; and, before they subsided, peripneumony came on, and he died. And how, it may be asked, does the author view this very common case? He casts a somewhat sharp reflection on the gentleman who had ordered a little mercury in one period of the case: he attributes the fever and dropsy to the mercury thus exhibited; and the pneumonic symptoms, which carried him off, to the falling out of some old rags from the broken panes of his windows! The case, we can venture to assure the author, is not without its parallel in the books of every surgeon in this town; and many a man in like circumstances has died, though his windows were glazed with the best crown glass.

We have, lastly, some fault to find with the author on the score of style and language. In page 77, for instance, we read of a respectable elderly gentleman, "who died after a very *tedious, lingering*, and *protracted* illness." Why would not one of these adjectives have done, without pressing its two synonyms into the service? Again, we find the author stating, at page 22, that "he could not obtain permission to examine the *morbid* condition of the *diseased* parts." Here again we have an adjective of supererogation.

But we must cease from this sort of desultory criticism, and enable our readers to judge a little for themselves, as to the merits of the work in question. Some of the peculiar opinions which Dr. Venables entertains, are more explicitly stated in the Preface than in any other part of the work, and therefore we commence with it.

"I have long regarded," says the author, "febrile and inflammatory action as the same in *essence*, and differing merely in the extent of parts occupied by the morbid action." This appears to us a very singular notion; nor can we imagine how the author reconciles it with the common phenomena of typhus. We should like to know what is "the part occupied by morbid action in this disease?"—or, rather, what part is not occupied by morbid action. If the author, as we conceive, means to aver that, in typhoid fever, every structure is occupied by morbid action of the same *essence* as inflammation, then we ask, how it is that so many recover from typhus, when so few recover of enteritis, where we know that only one structure suffers? The next page of the Preface presents us with another singular notion of the author. He tells us, that variola, measles, and typhus, are all considered as diseases of debility:—we presume he means (for the context admits no other meaning,) by the generality of practitioners. Now we should like very much to be informed of the names of a few of the leading practitioners who support the doctrine that measles is a disease of debility, or in any degree upon a par with typhus. With regard to small-pox, the case is even still stronger; and really we think Dr. Venables has formed a very poor estimate of the acquirements of modern practitioners, when he considers them as ranking small-pox and typhus in the same order of diseases. If the author had confined his objections to the employment of the term *disease of debility*, we might, very probably, have been disposed to agree with him. It is, in truth, as far as we can judge, a foolish unmeaning term: it apparently implies that there are *diseases of strength*,—or, in other words, diseases in which the strength of the body is increased; the very mention of which is repugnant to common sense.* All diseases then are, in a certain sense, diseases of debility; but there is a vast difference between the debility attending small-pox and that attending typhous fever, and no practitioner that we know of ever thought of confounding them.

The volume is divided into two parts. The first consists of the details of twenty-one cases, each followed by some clinical remarks, much after the manner of Dr. Crampton's Report on Dropsies; which, indeed, our author professes to follow. A few of these cases are curious and interesting; but by far the greater number are of every-day occurrence, and by no means merited the honor of publication. As an instance of the comparatively small acquaintance which the author has with the views and opinions of his professional brethren, we quote the following from the very first page of his Clinical Report:— "The sense in which I shall use the term pathology, differs in some degree from that in which it is usually accepted."— "Pathology I use as expressing the doctrine of those morbid or deranged operations of the economy, by which organic changes in our mechanism are affected."—"I consider pathology as opposed to physiology,—the former expressive of the doctrine of morbid, the latter of that of healthy, function."— And now, in the name of common sense, what does any other person mean by pathology than just this?

We give the following as a good specimen of the general character of the cases here recorded, and of the style of the author's clinical remarks. We select it, too, as being the shortest we could find, and as affording a favorable impression of the mode of practice which he pursues.

"Case XV.—Eliz. Smith, aged fifty, works at spinning: a poor, emaciated old woman. Dispensary.

"Nov. 14th, 1823.—Pyrexia; hurried respiration; pulse hard, frequent, and small; thirst; cephalæa, with slight cough, but much more

* We conceive that the increased muscular power, sometimes perceptible during the excitement of mania, can scarcely be admitted as an exception.

troublesome at night; bowels free; urine plentiful, turbid, with a kind of cloud in it; was not examined by heat. Abdomen swollen, with a feeling of distention, which has been increasing for several weeks past. Finds her clothes becoming too tight; œdema of the feet and legs severe, especially on the right side.—℞. Pulv. Digitalis gr. vj. Pulv. Ant. gr. xx. Ext. Hyoscyami gr. viij. M. Ft. pil. no. xij.; st. j. sing. noctibus.—℞. Sulph. Magnesiæ ℥ss. Carb. ejusdem ʒss. Aceti Colchici ʒij. Aquæ font. ℥vj. M.; st. coch. j. amp. ter in die.

"19.—Urine increased very much in quantity, but the patient rapidly emaciates; bowels free; pyrexia continues; giddiness; nausea pulse slower, but hard and thready; cough, ascites, and œdema, nearly as at last.—Mitt. sanguis ad ℥viij.* Omitt. pilulæ.

"21.—All the symptoms relieved; bowels constipated.—℞. Pil. Aloës c. Myrrhâ ʒss. Calomel gr. x. M. Ft. pil. viij.; st. j. alt. noctibus; cont. Mist. diuretica.

"Dec. 1.—Ascites much relieved; urine plentiful; œdema of the right leg still severe; bowels free.—Mitt. sanguis ad ℥viij. cont. mistura.

"3.—Cough severe; dyspnœa, with hurried respiration; fever; pulse firm, hard, and wiry; thirst; foul tongue; pain on pressure of the abdomen, which is enlarged; œdema of the legs increased; urine scanty and gelatinous, and coagulates by heat. Blood, drawn on the last occasion, did not buff. Attributes the increase of the symptoms to cold she caught going home.—Mitt. sanguis ad ℥x.—℞. Calomel gr. vj. Pulv. Ant. gr. xx. Confect. Opii q. s. ut ft. pil. viij.; st. j. tertiis horis. Ht. item pil. aperient. no. viij.; st. ij. sing. noctibus, aut pro re natâ.

"4.—Cough and dyspnœa relieved; respiration not so hurried; fever abated; pulse softer and less frequent; skin softer and cooler; abdomen diminished, and œdema less; urine sufficient. Blood buffed and cupped, with a dense, thick serum.—Cont. med. ut suprà.

"5.—All the symptoms much relieved; œdema has nearly disappeared.—Perstat usu remediorum.

"From this time she continued to improve; the ascites and œdema of the legs speedily disappeared. She took a little infusion of cascarilla as a tonic, and was discharged perfectly cured on the 28th January, 1824.

"*Observations.*—The exciting causes of the dropsy in this case I could not well ascertain; she was after what women term 'the turn of life.' There was, it will be observed, pyrexia from the first; but whether this pyrexia arose from cold, indigestion, or what other source, I could not well learn. There were no signs of local inflammation at the first application, but fever was present; I therefore determined to try what effect simply exciting the urinary organs might have upon the disease. In the report, both upon the present occasion and upon several others in the course of this history, the urine was not only plentiful from the first, but was still further augmented by the use of diuretics; but yet the dropsical symptoms remained unabated. Indeed, one circumstance was too obvious and too important to escape observation,—namely, the emaciation. The increased exhalation being carried off by the kidneys, whilst the cause of it was not removed, produced the

* The blood did not buff, but the serum was thick, dense, and gelatinous.

emaciation. Dr. Philip mentions the history of a glutton, who daily consumed an almost incredible quantity of food, yet he rapidly emaciated; the sustenance, as it were, passing off by the skin and kidneys. Indeed, I fear the reliance solely upon diuretics in active dropsies, has often produced diabetes. Dr. Blackall thinks that diabetes and dropsy are very nearly allied to each other; and I feel very much disposed to agree with him.

"There evidently was no organic disease, and therefore the complaint must have depended upon excitement of some kind, as we may presume from the active means which relieved the symptoms. Whether this excitement rose solely from the febrile state of the system, it is not for me to decide; I have given a true history of the case, let the facts speak for themselves." (P. 85—89.)

The general results of the twenty-one cases here detailed, may be classed as follows:—

| | |
|---|---|
| Cured . . . . . . | 14 |
| Relieved . . . . . | 3 |
| Died . . . . . . | 4 |
| | 21 |

But it must be borne in mind, at the same time, that many of those belonging to the first class were very slight: take, for instance, Case xvii., which we think would hardly have been set down as a case of dropsy by other practitioners.

We have already laid before our readers a sketch of the principal objects of the author in this publication; but it may, perhaps, be satisfactory that they should be given more at length, and in his own words:—

"From the foregoing Report, I am fully authorised in deducing the following inferences:—

"1. Dropsy is *(more)* frequently an active disease.

"2. When active, it is generally complicated with a pyrexial or inflammatory state of the system.

"3. When dropsy depends upon inflammatory affections of the viscera, the operation of these affections is most frequently indirect; namely, through the fever which commonly accompanies such diseases.

"4. Dropsy may arise as the termination of acute and subacute inflammation in the serous membranes, or in the cellular structures.

"5. Dropsy, in a very severe degree, may exist independently of any organic disease.

"6. When the cellular structures, or serous membranes, &c. are weak, either naturally from mal-conformation, from disease, or mechanical violence, the excitement of the economy, during the earlier periods of pregnancy,* may lead to a dropsy of the weakened parts, which, if

* "Of course, excitement of any kind will, under the same circumstances, lead to similar results. But pregnancy being a natural process, the excitement generally attending it would be suffered to pass unobserved and unheeded; whereas a much inferior degree of excitement, arising from an unnatural or unusual cause, would obtain serious attention. I therefore thought it necessary to notice specifically the probable consequences from pregnancy."

neglected, will, under unfavorable circumstances, become alarming and inveterate diseases.

"7. When dropsy exists in combination with an excited state of the system, or with an inflammatory affection of any organ or texture, antiphlogistic measures, especially blood-letting and antimonials, form the most rational and effectual means of cure.

"8. A reliance on diuretics in active dropsies serves merely to debilitate the system, without curing the disease, and may even lead to diabetes.

"9. A coagulable state of the urine in dropsy generally indicates the necessity of blood-letting; but the converse of this proposition does not hold good, and the non-coagulability of the urine does not necessarily prohibit venesection." (P. 122—124.)

We leave those of our readers who are familiar with the works of Blackall, Crampton, and other recent authors, on the subject of Dropsy, to say whether these "inferences" from Dr. Venables' cases add much to their knowledge of its general pathology. In the mean time, we pass on to the consideration of what the author calls his "Appendix," by which is to be understood a Dissertation, of about an hundred pages long, on the Theory and Treatment of Organic Diseases in general. From the constant recurrence of the name of Dr. Wilson Philip, and of his Treatise on Indigestion, a careless reader might be induced to suppose that this was a sort of review of that work. Dr. Philip evidently stands very high in the author's estimation; and, though we are far from thinking that his confidence in him is misplaced, still we would rather have had more of Dr. Venables' own views. However, we shall find enough to fill the remainder of the space which we can afford for the examination of this volume.

The very first point of doctrine with which our author sets out, appears to us to be in open defiance to commonly-received opinion, if not to common sense. Dr. Philip had remarked, that the perfect restoration of the function of an organ showed that its mechanism, *if disordered at all*, had only been so *temporarily*. Dr. Venables goes a step beyond this, and confidently maintains that every functional disorder, however slight, is attended with a corresponding change in its structure; "for to think otherwise," says he, "is to imagine an effect without a cause." Now, as we are no materialists, and have an old-fashioned notion about us, that the principle of life and of activity in organised beings is something not *belonging to*, but *superadded to*, their organisation, we take leave to differ from Dr. Venables. If a man, sitting down to dinner with a good appetite, hears of a sudden a piece of unwelcome news, he loses all appetite. If his mind is subsequently set at ease, his appetite returns. To imagine that the *structure* of the stomach has undergone any change in the interval, appears to us a most unwarranted, and withal most cumbersome and unphilcsophical, supposition.

Dr. Venables, as we before remarked, lays no small stress on a subdivision of disease into three stages. His words are—

"The animal mechanism may be considered susceptible of three degrees of change: the first consisting in such a change as has been generally named irritation,* and which Mr. Abernethy has termed *disorder;* the second degree comprehends those changes which are the result of the less severe inflammatory affections, and which, perhaps, may be termed *disease;* the third change includes those alterations which render the structure itself worse than useless, for it becomes a perpetual source of irritation, and, generally speaking, ultimately proves fatal." (P. 147.)

The author has obviously borrowed this notion from the classification of the stages of indigestion given us by Dr. W. Philip. The theory is a very pretty one on paper, but we doubt very much its application in practice. No allowance is made for the natural changes of structure consequent upon the advance of life,—the obliteration of some vessels, for instance, and the increasing disposition in others to deposit ossific matter. These, and several other changes of structure, we believe to be totally independent of either functional disorder or inflammatory disease.

The general principle laid down by the author, as regulating the theory of organic disease, is perhaps best stated in the following paragraph; to the justness of which, upon the whole, we are fully disposed to assent.

"In fine, from the different opportunities which I have had of inquiring into the history, and witnessing the progress of secondary diseases, and the termination of these in organic affections, I feel disposed to support the view which I have just now taken; and to attribute them to the fever which prevails in the system at large, operating prejudicially upon the less excitable parts of structure; and that the fixation of disease of this nature is determined by the comparative structure and strength of the affected part itself. We have too long been in the habit of regarding such fevers as sympathetic or secondary to the local affections; whereas it is equally reasonable, and in many instances much more consistent with the phenomena, to look upon the fever as the primary affection, and the local affections, and subsequent disorganisation, as the effects of febrile excitement prevailing generally, but unequally, through the system." (P. 177.)

---

* "Irritation merely expresses the phenomena or results of such a change We as often say 'there is great irritation of the bowels,' as 'that the bowels are in an irritable state.' We are much in need of a proper term to express this state. The term 'disorder' has been too general in its application to be now limited. It is no matter what term we use to express an idea, so as we limit its signification to the expressing of that idea."

In discussing the treatment of disease, Dr. Venables shows considerable talent. We were very much gratified by his animadversions on the tonic and stimulant treatment so often pursued in the first stages of indigestion. "The febrile form of that affection," says the author, (page 181,) "I have frequently seen induced by stimulating remedies exhibited in too great abundance, to restore the languid powers of the stomach; and if, in that state of the disease, such treatment be solely relied on, it will as certainly induce local inflammatory action, even where no traces of it, of the slightest kind, had previously existed." He goes on thus to express himself:—

"Patients labouring under febrile dyspepsia are generally oppressed with a languor and despondency which renders them extremely miserable. A stimulating treatment is calculated to intoxicate the senses, and render them insensible to every feeling of distress. Nay, even the spirits may be thus exhilarated, and a temporary respite obtained; but it proves an interval of repose, from which the patient awakens no way refreshed. He soon finds that there has been no real suspension of his sufferings, which now again press upon him with aggravated severity. Similar, but more powerful, means are now resorted to, and each successive effort is followed by still more disastrous consequences.

"Local inflammations now begin to show the peculiar tendencies of the habit; and fortunate will it prove for the patient, if the inflammatory manifestations show themselves in such a form, or appear in parts where their character cannot readily be mistaken. It has often happened that pains and tenderness, the consequences of internal inflammatory action, have been looked upon and treated as nervous, till the total disorganisation of the part has left nothing whereon to doubt, nor any thing to hope. In the febrile form of dyspepsia, before we attempt to arouse the vigour of the digestive organs, we must first reduce the inflammatory tendency. It may be observed of tonics and stimulants in general, that whenever they excite fever they are hurtful; and their exhibition under such circumstances should be suspended, till this tendency has been reduced." (P. 182, 183.)

We shall not stop to discuss the different measures recommended by the author, with the view of arresting the two first stages of disease, (or, as he, rather quaintly we think, terms them, changes in the animal mechanism,) because, though there is much good sense in them, there is no novelty. If we were to make any exception, it would be in favour of that part of the work (page 211,) which is occupied in recommending issues and setons, for the purpose not merely of *lowering*, but of *equalizing*, the circulation. We highly approve of this practice, and concur with the author in thinking that the best effects may often be expected from it.

And this brings us to the last object of the author's inquiry,—viz. the treatment of the third stage of disease, or that in which actual disorganisation has taken place, leading in its turn to fresh irritation. He begins by stating that, "as the inflammatory tendencies are not now subdued, and as the same unequal excitability continues, we must still be cautious in the administration of tonic and stimulant remedies." He then goes on to show that, while, in the two first stages of disease, our main object is to control and regulate *deposition*, so, in the third stage, our endeavours must be to awaken the dormant powers of *absorption*. For this purpose he advises—what physicians have advised, in similar cases, for centuries past—mercury. Having discussed the deobstruent virtues of this metal at some length, he enters upon the consideration of iodine, and devotes the ten last pages of the book to an examination of its compounds, and of their effects on the animal economy.

If we can thoroughly rely on the accounts of the virtues of iodine which the author brings together, we are certainly indebted to the chemists for a very powerful and valuable drug. "To deny," says the author, (page 228,) "that iodine possesses extraordinary powers in scrofula, would be to deny a fact which may be tested and proved, and which every day's observation and experience fully confirm." We are certainly not going to place ourselves in so awkward a predicament as the author here adverts to; but we would merely inquire, for information sake, where "the facts" illustrating this most interesting truth may be met with, and whether the practice of any of the great public hospitals in London or elsewhere bears the author out in an assertion so confidently put forth? We have ourselves tried the hydriodate of potass in the strumous swellings of children, in a considerable number of cases, but we regret to say without satisfactory results.

If mercury be so good a thing in organic disease, and iodine not much inferior, it was a very natural supposition that their union would give us a *tertium quid*, better than either. "The idea no sooner occurred to me," adds Dr. Venables, "than I instituted a number of experiments to determine the most advantageous mode of preparing,* as well as of ascertaining, the properties of the mercurial iodides." The details of these experiments he does not give, which we confess we very much regret, but their general results are thus announced:—

"Suffice it to say, that I succeeded to the full extent of my expectations on the first point,—that is, increasing the energies of the remedies by combination; and that I partially succeeded in the second. As far as regards the second object, depriving the remedies of their objectionable properties, it is not completely effected by their union. The combination is apt to excite the force of the heart and arteries, and so to increase the momentum of the circulation. Hence, when

* See Dunglison's Appendix to Magendie's Formulæ.

fever, or any marked tendency to fever, prevails, the diathesis wherein this tendency consists should be corrected, before the mercurial iodides be exhibited. It frequently happens that, where no febrile symptoms were observable before the exhibition of the iodides, some time after the administration a febrile tendency prevails, and the manifestations sometimes run very high. Immediately on the appearance of such symptoms, we should desist from the farther use of the remedy, and institute those means which experience has proved most efficacious in subduing fever." (P. 229, 230.)

The author next introduces to our notice the hydriodates of zinc and iron, (formed by decomposing a solution of the metallic sulphates by one of an alkaline hydriodate,) which, he says, are mild and gentle tonics, particularly the latter, "admirably adapted to fulfil the indications laid down for the cure of fully-formed organic disease."

We are promised (page 106,) a more full account of the virtues of these metallic iodides, "at a more favourable opportunity." Until then we shall defer offering our own suggestions. We cannot help remarking, however, that in the only two instances that appear in this volume, in which iodides were given, (Cases iv. and xviii.) the patients died, without the slightest apparent effect being produced by them. This, we must confess, does not hold out to our sober judgment much encouragement; but we are very willing to "live and learn."

We here take our leave of Dr. Venables. If, in the course of our remarks, we have had occasion to animadvert in terms which he may deem harsh, on some of his opinions, we are, on the other hand, not insensible to the honourable zeal which he has displayed in the cause of his profession, and which it would be uncandid to pass over without the tribute of praise to which it is so justly entitled.

---

Art. II.—*Opinions on the Causes and Effects of the Disease denominated Tic Douloureux; deduced from practical Observations of the supposed Origin, in lateral Pressure, Distortion, or undue Contact of the Teeth, but more particularly those nearest the Maxillary Sinus, and thence conveying its distressing Sensations to the more distant Extremities of the System. The whole illustrated by three Lithographic Engravings, with annexed Cases, confirmatory of the Opinions and Suppositions; as also a peculiar and easy Mode of Ascertainment and Cure.* By Charles Bew, Surgeon-Dentist to his Majesty and Royal Household, and also to their Royal Highnesses the Duke and Duchess of Clarence.—T. and G. Underwood, London, 1824.

People in general are very cautious how they trust a gold watch or a repeater in the hands of an inferior or country workman, but they do not scruple to trust that *simple* machines, the human body, to any one who puts up a board to say "all disorders rectified, and exhausted constitutions neatly mended here."

Lady Mary Wortley Montague, in one of her admirable Letters, has attempted to account for the extraordinary facility with which her countrymen are duped by the most ignorant quacks, and says, very truly and very ingeniously, the English are more easily infatuated than any other people by the hope of a panacea; nor is there any other country in the world where such great fortunes are made by physicians. I attribute this to the foolish credulity of mankind. As we no longer trust in miracles and relics,* we run as eagerly after receipts and doctors; and the money which was given, three centuries ago, for the health of the soul, is now given for the health of the body, by the same sort of people—women and half-witted men. Quacks are despised in countries where they have shrines and images. True it is that medical quacks do not flourish in catholic countries; the business of superstition is there entirely in the hands of the priests, and the comparative poverty of the people prevents them from applying to any but saints and relics for the exercise of their faith and hope in most diseases. The government of these countries facilitates the acquisition of medical knowledge, by establishing schools in different parts of the kingdom; and prevents the people being duped by ignorant and wicked pretenders, by arresting all those who publicly set forth their false pretensions. Any scientific discovery is immediately seized by some of the numerous adventurers in this country, who prey upon the miseries and follies of their fellow-creatures. Electricity and galvanism, magnetism, oxygen, and all the endless varieties of elastic fluids, have been most ingeniously and successfully enlisted into the service, and tortured to the purposes of quackery; and our newspapers afford the highly prolific means of making known the various properties and cures ascribed to those agents, administered under various forms, and boasted of with an endless perseverance.

The above observations on some of the effects and the seductive allurements of empiricism, have been reluctantly called forth by our perusal of the pamphlet—the very extraordinary pamphlet, whose title is at the head of this article. We were induced, by the high pretensions contained in the title-page, to give an attentive as well as an early reading of its contents; and our duty to our readers, and that even-handed justice which we arrogate to ourselves some merit for dispensing in the most impartial manner, and in the most accurately-balanced scales,

---

* What would Lady Mary say, in these wonder-working days, were she acquainted with the miracles of Dr. Hohenloe and Co.?

compel us to exercise a degree of critical severity, which, we trust, is never called forth but upon the most imperious and palpable occasions. Towards the author, a Mr. Charles Bew, dentist at Brighton, we disclaim every feeling of a personal nature, as we declare that, prior to the appearance of his pamphlet, his name even had never been heard by us; and we here protest our total and unaccountable ignorance of the existence of this very loyal individual, surgeon-dentist to his Majesty and to their Highnesses of Clarence, and breathing the royal and salubrious air of Brighton. (One might see, from the Dedication, that he had flourished in the neighbourhood of the court.) Of this ignorance, we take shame to ourselves in making the ungracious confession, especially as we find him author of some opinions on the Teeth, which, not being known to us, have of course escaped our critical attention; although he informs us that they have met with "general approbation and flattering success, in this country and on the continent." Our humble confession, however, will, we trust, have the effect of exonerating us from the imputation of any personal feeling towards Mr. Bew.

The lithographic print prefixed to the title-page is intended to delineate the ramifications of the different nerves distributed to the face. In a practical point of view, it is not of any very great importance to specify the inaccuracy of position and situation given to every branch; the ignorance of anatomical information displayed in this plate; and the errors into which the study of this print will lead every inquirer into the distribution of the nerves chiefly suffering under neuralgic tortures. The branches of the seventh pair are allotted to the fifth; their origins, distributions, and emerging points, are most grossly mistaken; and it is our bounden duty to caution our readers against forming any anatomical ideas of those nerves from this *mis*-representation. The consulting and copying any one of the excellent and numerous plates descriptive of the nervous system, might have been the means of sparing us the trouble of making, and Mr. Charles Bew the pain of enduring, these necessarily severe, but just, remarks.

A two-fold Dedication to his Majesty and the Duke of Clarence, given in bad grammar and bad taste, prepare us for the numerous practical errors and gratuitous assertions pervading the whole body of this singular production: were they, however, errors affecting only the personal reputation of the author, our critical animadversions might be dealt out with a more lenient and a more sparing hand; or we should have suffered the work to fall into the oblivion which awaits it: but when we reflect that the comfort of numerous individuals may be connected with the effects produced by this book upon unprofessional readers, the due exercise of our duty and privilege must bear away our accustomed disposition to extend leniency to those whose intentions are honourable, although their judgments may be erroneous.

The main and leading object of Mr. Charles Bew's book is an attempt to show the intimate connexion of neuralgia of the face with the nerves of the teeth nearest in situation with the maxillary sinus, "or that portion of the upper jaw devoted to the disposition of the four grinding teeth, anatomically denominated the two bicuspides and two molares. Unfortunately, attacks in the teeth, like fits of the gout, excite little or no sympathy, and less consideration, in those who are happily strangers to the severity of their visitations: indeed, the sufferers themselves, demonstrating to proof the self-evident axiom that pleasure is only the absence of pain, on the subsiding of pain either in the teeth or the extremities, because they can comfort themselves with the deceitful hope that the paroxysm may not be repeated, feel little solicitous about the rise and progress; and thus, by not ascertaining the cause, neglect the only rational or probable means of preventing a return." (P. 3.)

Our limits denying us the power, and the weakness of our antagonist taking from us all inclination, of entering fully into the arena of argument against the absurd propositions of Mr. Charles Bew, we shall confine our very few remarks to matters of fact and of well-tried experience, deduced from our own observations on tic douloureux, and from the judicious and accurate representations of the numerous authors on this subject, who have of late devoted a very considerable share of time and attention to the investigation of this important part of pathology, and whose valuable opinions have been conveyed to the public, in a great measure, through the medium of our Journal.*

"To enumerate all the cases of tic douloureux which have come under my consideration, and have been crowned with convalescence by the peculiar *modus operandi* which it has been the chief object of this production to promulgate, for the advantage of practitioners and the public in general, would fill a volume; and, after the fatigue attendant, the reader would only arrive at the information my inferences have heretofore induced, that the disease, the subject of this opinion, is produced by undue pressure in and on the teeth, causing excitation or irritation of the dental branches of the nerves, whose extremities we find infinitely diffused on the face and parts usually affected. In the selection of cases, therefore, I shall be desirous to describe such as, varying somewhat in situation and *originality of sensation*, still had their cause and effect traceable to teeth and nervous irritation, and thus entitled to the denomination of tic douloureux." (P. 49.)

* See also Mr. Ranking's paper in the present Number.

Our readers, already acquainted with Mr. Charles Bew's supposition of tic douloureux having its origin in the teeth, will require from our hands but few arguments to convince them of this gentleman's erroneous pathology and injurious practice: with his motives we have nothing to do, although it is impossible to mistake them.

The very ample experience of Mr. HUTCHINSON in the treatment of neuralgia,—his excellent observations on the origin, cause, and management of this disease, confirmed by the practical remarks of many of our most esteemed and respectable practitioners, among whom we may enumerate Sir HENRY HALFORD, Sir ASTLEY COOPER, Dr. ELLIOTSON, Mr. A. T. THOMSON, Dr. BORTHWICK of Edinburgh, Dr. MARSHALL HALL, Dr. STORER of Nottingham, and a long list, which we could without difficulty select, are most satisfactory testimonies, in our estimation, of the cause and origin of neuralgia *not* having their existence in the teeth. That these important organs of our masticatory system generally sympathise with the disturbed and frequently diseased state of the highly sentient extremities of the nerves of the face, no one will presume to deny. Their proximity and alliance with the parts affected render their participation in the distresses of their neighbours a matter of the greatest probability. In the numerous instances of neuralgia of the face, which have occurred in the course of our own experience,* in many of which suspicious teeth had been previously removed, *not the most remote relief was ever obtained by the operation of extraction. On the contrary, this additional cause of irritation had added a hundred-fold to the misery of the sufferers.* This is all the answer we shall make to the assertions of Mr. Bew; and, when we assure our readers that it is a plain statement of what we have ourselves seen, we trust the answer will be deemed sufficient. In the last interview we had with that excellent physician, the late Dr. PEMBERTON, he declared to us that he had endured great and manifold increase of pain, since he had submitted to the removal of teeth, and the division of the facial nerves.

We shall take leave of Mr. Charles Bew, by entering our most formal protest against his language, his grammar, his orthography, his theory,—and, what is of more vital importance than all the rest in conjunction, the most injurious and destructive practice which he recommends.

---

*** This critique is from the pen of a Correspondent, who has had ample opportunities of witnessing this formidable disease, and who consequently speaks from personal experience.—EDITORS.**

ART. III.—*Report of the Epidemic Cholera, as it has appeared in the Territories subject to the Presidency of Fort St. George. Drawn up by order of the Government, under the Superintendance of the Medical Board.* By WILLIAM SCOT, Surgeon, and Secretary to the Board.—Madras, 1824; folio, pp. lxxii. 292. *With an Appendix; containing the Sick-Returns of the Army of Fort St. George, from the year* 1815 *to* 1821, *inclusive, exhibited in a series of fifteen Tables; also Diurnal Tables of the Progress of Cholera, in six Parts; Tables of the Movements of the Troops, and Meteorological Tables, in seven Parts, from* 1815 *to* 1821.

[Concluded from page 80.]

WE now have arrived at the Method of Treatment.

Nothing more distinctly shows the intractable nature of a disease, than the recommendation of a multitude of specifics; and this has been strikingly the case in cholera, the progress of which, at a certain stage, has sometimes been arrested by the most opposite, or apparently the most trifling, remedies; and it was observed by practitioners, that, after the first appearance of the disease, the remedies, which they had formerly found successful, were afterwards totally unavailing. The general indications of cure appeared to be two,—namely, to moderate inordinate action, and to support or restore depressed actions; but, as it was soon found that these indications could not be fulfilled by the remedies prescribed, a great diversity of opinion and practice followed. This perplexity was increased by the different prevailing types of the disease, and it is only of late that the necessity of its treatment upon general principles appears to have been acknowledged.

*Opium* stands first upon the list of those medicines which were employed to calm gastric irritation and subdue spasm; and, when given at an early stage of the disease, its effects were decidedly successful, and this more particularly among the natives, whose simplicity of living renders them less liable to complicated forms of disease. The appropriate dose of this medicine it appears not so easy to ascertain. From eighty to one hundred drops of the tincture, and from two to four grains of solid opium, was the dose most usually exhibited; but, as we may suppose, with many practitioners the quantity employed was sometimes much great greater, and in some instances not so great; but the first doses were usually given in the liquid form, and the subsequent ones in the form of pills. It is no imputation upon the virtues of opium to acknowledge that it has often been given in vain, and that many cures have been performed without it; but we may, in general terms, remark, that those cases may most confidently be trusted to opium, in which the stomach and intestines are chiefly affected, as indicated by vomiting, pain in the epigastrium, or purging. Upon the whole, we are not to consider opium as a specific in cholera, but as an auxiliary of the first importance.

Of *ether*, *ammonia*, *musk*, *camphor*, *castor*, &c. we may shortly remark, that, in conjunction with opium or calomel, many of these remedies have been found useful; but the period at which they can be safely employed is extremely limited: after a certain time, the stomach appears to be no longer sensible of their action.

*Wine* and *spirits* have been exhibited very extensively in this disease. The same remarks apply to the exhibition of these stimuli as to that of opium; and, with reference to this practice, Mr. Scot very judiciously observes—"The extent to which they should be given has not been sufficiently considered, with a reference to the actual state of the patients, and to the laws of excitement, as far as they are generally understood and admitted. If the collapse in cholera be the effect of a direct diminution of the capability of an organ to be affected by stimuli, we should commence by doses large in proportion to the supposed degree of this diminution; and we should decrease them according to the progress made in restoring excitability: but, if the collapse be merely owing to a want of natural stimulus, the doses should be small in the commencement, and increased gradually."

Some few practitioners, we are told, abstained entirely from the use of ardent spirits, from a belief in the inflammatory nature of the disease: but their practice does not afford any justification of this view.

In administering *calomel* for the cure of cholera, different indications have been followed: it has been given as a mode of quieting irritability, of emulging the biliary vessels, of restoring the balance of the circulation, and sometimes without any reason at all. The result of experience, however, shows that this medicine does not possess any specific virtue in the cure of this disease: the success of those practitioners who did not employ it has been fully as great as those who did, whilst the arguments brought forward against its *early exhibition* appear conclusive; and it is only when a favourable change has taken place, and the ordinary functions are renewed, that calomel is clearly indicated.

*Blood-letting* next claims our attention, a remedy, as it would appear, of the first importance; and yet, observes Mr. Scot, it requires no common effort of reasoning or reflection to arrive at the conclusion, that, when the powers of life appear depressed to the lowest degree, the pulsation of the heart all but extinct, the natural heat of the body gone, and the functions of the system suspended, the abstraction of blood might yet prove a remedy against a train of symptoms so desperate. Bleeding was at first employed in cases where there was much spasm; and, dissection showing often a loaded state of the vessels of the viscera, it was adopted also to remedy this condition. When syncope was brought on by venesection, it was generally favourable; but the information as to the manner in which it was performed, or the quantity taken away, appears to be defective, though its utility is most generally and unequivocally admitted. It is remarked that fatal collapse has sometimes followed bleeding, but more frequently after the abstraction of a *small* quantity of blood; for, if the evacuation is persevered in until the effects reach the internal vessels and the heart itself, then the circulation seems to be freed from an oppression which impeded its functions.*

Such is the theory which has been adduced in support of blood-letting in cholera; and, if true, the supervention or presence of collapse, so far from deterring us from going on, should only be regarded as additional reasons for renewing our efforts to get blood. This is supported and confirmed by many apposite quotations from the Reports, which to us appear perfectly conclusive on this subject; and the only difficulty attending the practice relates to the almost impossibility of obtaining a sufficient quantity of blood: but the operator must call to his aid the use of frictions, hot water, internal stimuli; he is not to be deterred by any immediate accession of debility, nor is he to be satisfied with a temporary amelioration of the pulse; for, if he can freely unload the internal vessels, he will probably save his patient; if he fails, he will most probably lose him. The principle is, that, in cholera, collapse is not the consequence of loss of blood, but that it is a condition only to be relieved by its abstraction. Nevertheless, we are taught not to rely solely on this remedy, but to use it in conjunction with others. The Medical Board have, in their circular letters, advised the exhibition of antispasmodics and stimulants *prior* to the use of the lancet. When general bleeding fails, topical blood-letting may be resorted to.

Of external remedies, the *warm* and *vapour baths* are first mentioned, but they were not found to afford the relief anticipated from their use; in the formidable cases especially (those of collapse,) no advantage was gained: nay, the patient, at the time that his skin was deadly cold, would frequently complain of a moderate degree of heat as scalding and intolerable.

*Dry heat*, by means of bags of heated salt or sand, have also

* It may be remarked, that the favourable opinion of the effects of blood-letting, as described by Mr. Scot, coincides with that of the author of a paper which appeared in this Journal a few months ago.

been occasionally employed with good effect, assisted by frictions of rubefacient and stimulant substances; but our author is of opinion that *sinapisms* have not been so extensively nor so early employed as they might have been.

*Vesicatories*, either of cantharides or of boiling water,—the application of *mineral acids* to the skin, over the region of the heart, or upon the abdomen, have often been employed with good effect, though the state of the skin, especially in an advanced state of the disease, renders their action very uncertain.

*Emetics* and *purgatives* are next mentioned. Of the former, the tartrite of antimony seems to have had its advocates, but latterly its employment has been abandoned. Mr. Scot seems to think that a combination of emetic substances with opium may be found more useful. With respect to cathartics, it would seem that their utility has its supporters, and is said to deserve further trial. Castor-oil, combined with laudanum especially, has latterly been used, in cases of natives, with very considerable success; but there is more difference of opinion as to the period when the purgatives ought to be administered,—some recommending them merely to obviate the sequelæ of the disease, whilst others prescribe them early. That medicine which does not produce a watery purging is considered the best. Infusion of senna, with gentian or ginger, the various cathartic extracts, the tincture of aloes, with myrrh and benzoin, &c. are recommended as the most appropriate.

Of the benefits derivable from *glysters*, there is no precise information afforded us.

The interdiction of diluents or drinks in cholera, was general in the first invasions of the disease, notwithstanding the urgency and intenseness of the thirst by which it is attended; but our author sums up his remarks upon this point by observing, that tepid, diluent fluids should be freely, and even largely, given in cholera, especially at the commencement of the attack, where the stomach is yet active; and that the experience of many practitioners warrants the safety of using drinks acidulated either with the mineral or vegetable acids. The administration of food resolves itself into two or three simple rules, rather to combine it with diluents in the form of sago, arrow-root, barley, or rice; and, in ordinary cases, we may begin to give nourishment, independently of these drinks, as soon as the disease appears to have yielded.

The general rules that follow are such as are applicable to convalescents in general, and we, therefore, need not repeat them.

It is of the greatest consequence in cholera to husband the patient's strength, and, therefore, all exertion of the muscles of voluntary motion should be avoided; a task of much difficulty, where the sensation of restlessness is so distressing.

We have thus gone through an examination of Mr. Scot's Report, compressing as much as possible the valuable matter it contains into the compass of a few pages, and only lamenting that our limits will not permit us to give any extracts from the many able Reports that fill up the remainder of this volume. The numerous valuable Returns and Meteorological Tables are, of course, out of our power to copy, and we can only refer our readers to the original work.

We shall close this long, but we hope not tedious, article with the following Return, evincing the extent to which this terrible disease affected the army of Fort St. George, from 1815 to 1822, with the ratio of mortality in each year. We may probably be induced, in a subsequent Number, to extract some particulars from the annexed narrative of the progress of the disease in the peninsula of India, which is in many respects highly curious and interesting.

*Tabular View of the Number of Cases of Cholera occurring in the Army of Fort St. George, from* 1815 *to* 1822.

| | *Europeans.* | | *Natives.* | | *Strength.* | | |
|---|---|---|---|---|---|---|---|
| *Years.* | *Admitted.* | *Dead.* | *Admitted.* | *Dead.* | *Europeans.* | *Natives.* | *Remarks.* |
| 1815 | 65 | | 37 | | 13,409 | 59,672 | |
| 1816 | 97 | | 92 | | 13,943 | 61,969 | |
| 1817 | 168 | | 114 | | 12,959 | 61,641 | |
| 1818 | 1,037 | 232 | 3,314 | 664 | 10,652 | 58,764 | |
| 1819 | 564 | 85 | 3,779 | 734 | 10,125 | 63,782 | |
| 1820 | 356 | 69 | 3,322 | 758 | 9,416 | 76,870 | |
| 1821 | 357 | 39 | 2,527 | 830 | 9,553 | 82,046 | |
| 1822 | 774 | 170 | 548 | 199 | 10,813 | 74,707 | |
| | 3,138 | 595 | 13,490 | 3,185 | | | |
| From 1818 to 1822, add | 526 | 100 | 2,340 | 550 | | | Not in the regular Returns. |
| Total .... | 3,664 | 695 | 15,830 | 3,735 | | | |

"The general returns for 1815 to 1817, do not exhibit the diseases from which casualties arose, and it is not known, accordingly, whether any, or how many, of the cases of cholera during these years terminated in death; but, in the course of the first four months of 1818, when seventeen cases of cholera took place amongst Europeans, no death ensued; in May, fourteen cases occurred, and nine deaths. Amongst the natives, during January and February, ten cases took place, without a casualty; in March, twelve cases and two deaths; in April, thirty-seven cases and thirteen deaths; in May, seventy-two cases, and twenty-four deaths. We may, therefore, conclude that the epidemic cholera furnished the first casualties amongst Europeans in May, and amongst the natives in March 1818; and that, prior to that period, the casualties from cholera, commonly called cholera morbus, did not exceed the usual proportions."

---

### DIVISION II.

### FOREIGN.

ART. IV.—*L'Art de prolonger la Vie de l'Homme.* Par C. F. HUFELAND, Premier Médecin de S. M. le Roi de Prusse; Chevalier de l'Ordre de l'Aigle rouge de second Class; Professeur de Médecin de l'Université de Berlin; Directeur de l'Academie Royale de Chirurgie Militaire; Médecin en chef de l'Hôpital de la Charité; Membre de l'Academie Royale des Sciences de Berlin, &c. Traduit de l'Allemand, sur la seconde Edition, par A. J. L. JOURDAIN, Docteur en Médecine de la Faculté de Paris, &c. &c.—Paris, chez Bailliere. 1824.

*The Art of prolonging the Life of Man.* By C. F. HUFELAND, first Physician to H. M. the King of Prussia; Knight of the Order of the Red Eagle of the second Class, &c. &c. Translated by A. J. L. JOURDAIN, M.D. of Paris, from the second Edition.—Paris: Bailliere, 1824; pp. 436.

THE work which we have now undertaken to review, may be said to teach two arts at least,—the art of prolonging a book, as well as that of prolonging life; although it must be confessed that we, on this side of the Channel, stand in need of little instruction in the former of these subjects. Professor HUFELAND is already well known as the author of so many books and pamphlets, that their very titles alone would fill some few pages; and, in composing the present volume, he seems to have aimed at rivalling the gentleman alluded to by Baron GRIMM, who wrote a book entitled "De omnibus rebus et quibusdam aliis."

As it is our intention, in reviewing M. Hufeland's work, to dwell chiefly upon that portion of it which bears upon the subject which he expressly intends to illustrate, we shall here only give the heads of a few chapters, in order to show that we have not capriciously, or without reason, made the above comments on this laboured production; for, after all, it is a pleasing and entertaining book, and being intended for the public at large, many of our author's flights and excursions may be pardoned; though, to the mere medical reader, who sits down to a work for the purposes of instruction only, it is a very heavy infliction to be obliged to pick out one or two new ideas from such a mass of extraneous matter. Our examples of unnecessary chapters are the following :—A chapter on the Duration of Life in Vegetables; one on that of Animals generally; a long examination of the different exploded methods of attaining Longevity, including the philosopher's stone and the grand elixir; a chapter on Imaginary Diseases; and a long chapter upon Poisons and Contagions: so that, in truth, it is not until the 281st page that we arrive at the real subject of the work, since it is obvious that the premature destruction of life by poison or pestilence has nothing to do with the art of prolonging it.

The first chapter which contains a brief history of the various delusions that have from time to time been practised upon mankind, with the view of protracting the period of his existence, is amusing enough, embracing a sketch of the history of animal magnetism, and of Graham's celestial beds; but it is entirely devoid of instruction to the professional man, and indeed deficient in novelty.

Of the second chapter, we shall only quote the title, in order to show how unprofitable a task it would be to analyse it. It is an attempt to explain the nature of Life itself, and it commences with questioning whether it be possible to penetrate the intimate nature of this *sacred flame*, and to learn to distinguish what feeds and what weakens it? We need scarcely say, that our author's labours terminate in enumerating the common phenomena of existence, and the agents by which it is affected.

We pass entirely over the third and fourth chapters, on the Duration of Life in Vegetables and Animals generally, and pause at chapter the 5th, which contains an account of the most remarkable instances of Human Longevity. In the commencement, M. Hufeland invades the province of theology, by endeavouring to explain the extraordinary longevity of the antediluvian world; and he imagines that he has solved the difficulty by adopting the theory of HENSEER, that their years were composed of three months only. However this may be, we find, from Abraham downwards, few difficulties of this kind to contend with; and our own times almost, and our own nation, afford us examples of life protracted to at least an equal degree.

M. Hufeland next enumerates the principal facts that bear upon this question as recorded by the pagan authorities, which he brings down to the present time; remarking, that but few, if any, instances of longevity are to be met with, throughout the records of history, among princes or potentates; whereas, among hermits, and those subjected to the most rigorous regimen in every way, a multitude of examples may be found. Philosophy also appears to have extended the gift of long life to its votaries in all ages, from Plato down to Newton. We are next presented with the histories of Jenkins and Parr, and other long-livers of Denmark, Germany, and France; and an extended, but not very clear, account of an English humorist, called Nobs, is then given, who is said to have lived to a great age, and whose daily promenades are mentioned at some length. But how the young folks of a certain *town in Canterbury* should be so very much amused with the oddities of an old man, who walked daily to Highgate and back again, we do not readily understand. M. Hufeland does not notice, and perhaps does not believe, the history of the black woman, Louisa Truxo, who is said to have been alive in Jamaica in the year 1780, aged 175 years. But the last example he records is extraordinary, and new to us: it relates to an inhabitant of the vicinity of Berghen, in Norway, who died in 1797, at the age of 160. This man preserved to the last moment the use of his senses and his understanding. The evening before his death, he assembled all his family about him, and divided his possessions among them. He had been married many times, and his eldest son was 105 years of age, and the youngest nine. In conclusion, our author affirms that the British islands appear to be most favourable to longevity, France and Italy less so. Greece maintains the reputation that it always possessed in this respect; but neither extreme heat nor cold would seem to be favourable to the life of man. Both Germany and Holland contain many old men, but few who attain extreme old age.

The sixth chapter is an attempt to generalise the facts recorded above, and to reduce them to certain rules; but how fatal such attempts generally are, even in the most skilful hands, is shown by our author's sixth aphorism, which begins thus:—"That which contributes most to prolong existence, is a certain uniformity of temperature: this is the reason why countries where the barometer and thermometer are subject to sudden and considerable changes, are never favourable to life;" and yet, in the face of this, we find that England, Scotland, and Ireland, stand pre-eminent in this respect,—a most palpable contradiction! There does not appear, in the remaining aphorisms of this chapter, any thing to excite particular remark, excepting that M. Hufeland notices, and insists upon our keeping in mind, that all those who have attained to a great age have been married. This applies equally to females as to males.

Having thus shown to his readers the materials with which he is about to construct the didactic portion of his work, M. Hufeland stops to give us, in his seventh chapter, a general view of the organs, faculties, and functions of man,—of the connexion between the material part with the soul, and other high speculations,—from which we learn that he is perfectly untainted with the modern doctrines of materialism; and anxiously explains his thorough conviction of the existence of an immortal and immaterial principle.

We should scarcely have been tempted to have made any extracts from the eighth chapter, which gives us, in the form of aphorisms, the conditions of the various organs of the human body necessary for the attainment of longevity, had not our author dwelt with more than usual earnestness upon the perfection of the generative faculties, as tending to lengthen the period of life; and the sum of what he says is as follows:—

1. The organs of generation having the faculty of extracting the most spiritual parts of alimentary substances, are at the same time so organised as to return into the blood the fluids purified and perfectioned in them; and they are therefore, as well as the brain, in the class of those organs most essential to the perfecting our nature. External substances would be of little use to us, (says our author,) unless we were possessed of organs capable of extracting and elaborating the most subtile parts, and returning it into the system under this new form, identifying it with our being.

2d. That which is capable of giving life, ought also to have the power of preserving it: the vital power is so concentrated in the seminal fluids, that the smallest quantity can call another being into life. Can there be imagined a better means for the restoration or conservation of the vital powers?

3d. Experience proves that the body does not acquire its utmost perfection until the generative organs are in a condition to produce this new species of fluid; which proves evidently that they are not destined only for the production of other beings, but are immediately and principally intended to exert an influence over the entire system, upon which they impress a character entirely new.

4. Men who are deprived of the organs of generation, are also deprived of all the advantages above mentioned.

5. Nothing exhausts the vital powers so soon, or so much, as the loss of the seminal fluid: nothing gives so strong a feeling and charm to the sentiment of existence, as an abundant provision of these fluids; so nothing inspires ennui, and a disgust of life, so completely as their exhaustion.

In the three following paragraphs, M. Hufeland asserts that there is no example on record of an eunuch who has lived to an advanced age; that those persons who have lived to the greatest age have all been remarkable for their generative faculties, which they have possessed to the latest period of their lives; and that, above all things, they have never abused these faculties, but have employed them in the most regular and orderly manner.

This chapter concludes with the description of a man destined for long life. The features of this portrait do not need enumeration, since they are such as are agreed on by all.

The ninth chapter we pass over entirely; every subject of which it treats is to be found either in the former or latter parts of the volume.

We have now arrived at the second part of M. Hufeland's work, which is entitled the "Practice of the Art." The first twelve chapters in this portion of the volume, from page 203 to 281, are devoted to the consideration of the different causes tending to *shorten* life: they appear to us only intended to *lengthen* the book. Their titles are—on Education ill directed; on the Abuse of the Pleasures of Love, and Onanism; on Excess of Mental Labour; on Diseases; Violent Death, and the disposition to commit Suicide; on Vitiated Air, and the excessive Population of large Cities; on Excess in Eating and Drinking; on the Affections and Passions; on the Fear of Death; on Idleness and Ennui, on Imaginary Diseases; on Poisons and Contagions; and on premature old Age. Now we do not mean to say that there are not many good remarks, and several entertaining stories, contained in this long space; but, as our business is expressly with the converse of all these several questions, we conceive it more profitable to pass them over, and commence our extracts with the second section, entitled "the Art of prolonging Life," the first chapter of which treats of the Health and Vigour of Parents; and here we shall find that being *well born* has a very different signification with M. Hufeland, than the great ones of the world would suppose,—for it means to be born with such a fund of vital energy, as to promise the greatest extension of time to our existence of which it is capable. For the attainment of this desirable end, the three essential objects to be considered are—the health of the parents, the moment of generation, and the period of gestation. The first condition is easily understood, and can hardly require a comment; the second would have been a rich treat to Mr. Shandy, had he fortunately been as long-lived as some of our author's examples: it is one of those speculations in which he would have luxuriated; and we are quite sure that he would not have let it escape in the space of half a page, as our worthy author has done. To be serious: however true M. Hufeland's remarks may be, they are such as must for ever be useless and unprofitable; and what he observes of the children of illicit commerce, has been before said in the vivid and beautiful language of the poet Savage:—

> "No sickly fruit of faint compliance he,
> But stamped in Nature's mint, with ecstasy."

We fear that our author's observations relative to the period of gestation, though many of them marked by good sense, will be little relished by modern fine ladies; and that, among the rest, he will find it very difficult to persuade *nervous ladies* that they ought not to marry.

The second chapter, on the Physical Education of Infants, is in every respect a good one, though we cannot say that it teaches *us* any thing new: on the continent it would appear to be more necessary, since M. Hufeland condemns the custom of some who, he says, through *Anglomania*, give their infants meat, wine, and beer, in order to make them strong. It is thus that we are misunderstood, and consequently misrepresented. To nourish infants with meat, wine, and beer, is not the practice in England; it is condemned by all good medical authorities; and we are quite sure such a custom little deserves to be cast as a reproach upon this country, which, with all its advances in civilisation and science, has faults enough of its own, without bearing the imputation of those which do not belong to it.

In speaking of the education of children from the age of two to fourteen years, there are two points which our author insists upon, and they are both deserving of attention. The first is, not to exercise the faculties of the mind prematurely; and the second, to prevent the disposition to onanism, which very often manifests itself at a much earlier period than people in general suppose. His methods for this purpose are rational, but such as would readily occur to any parent or guardian, consisting of light vegetable food, plenty of exercise, sleeping on a mattress, loose dressing, so as to prevent any pressure or friction on the parts of generation, avoiding every gesture or discourse likely to lead to sexual emotions, &c. &c.

A few lines are devoted to the recommendation of strong exercise during youth, preceded by a remark, that all those who have attained a very advanced age have undergone great labour and fatigue in their younger years; and this is the substance of the third chapter.

Chapter the fourth is one of the most remarkable in the volume; it will be a very startling one to the youth of this forward and precocious age; but it is, in our opinion, filled with very valuable matter, and is equally honourable to the heart and understanding of the writer. It begins with asserting that there was a time when the Germans never thought of the commerce of the sexes till the twenty-fourth or twenty-fifth year of their age, and without experiencing any of those evils attributed in modern times to continence; so far from it, they acquired a degree of vigour that astonished the Romans themselves. With the peculiar views which our author entertains of the important part which the seminal fluids perform in the animal economy, we are not surprised at the earnestness with which he recommends a rigorous abstinence from the pleasures of love, until a permanent connexion is formed by marriage; and we are therefore prepared for the applause which he lavishes upon the days of chivalry, and the high and romantic tone which pervades the whole chapter.

M. Hufeland sums up in a few paragraphs the ill effects of premature and illicit indulgence; and then offers some rules, by the observance of which chastity may be preserved, and which consist in moderate diet, strong exercise, application to abstract sciences or to business, avoiding every thing that tends to inflame the imagination or excite sensuality, and by reflecting upon the dangers and consequences of libertinism. Our author truly remarks, that a most essential rule is to avoid the first error of this kind, since no propensity is so much increased by indulgence.

We again repeat, that, however little this chapter may be suited to the feelings and modes of thinking of the youth of the present day, it is honourable to the writer; and that he who has strenth of mind to follow his precepts will, in after life, reap a rich reward in the possession of a clear conscience and a sound constitution.

We have praised our author's last chapter, and with sincerity. His next strain is in a style still more elevated; and in these calculating times, where the burthens of matrimony cannot, and indeed ought not, to be lightly undertaken, we should be almost afraid of making many extracts from this part, which is entitled "on the Happiness of Marriage."

With respect to Sleep, our author thinks that six hours is the least, and eight the greatest, length of time which should be devoted to this refreshment. There are no new remarks in this chapter. M. Hufeland repeats the trite observation, that all the long-lived have been in the habit of rising early.

We shall pass rapidly over several successive chapters, treating of Exercise, Travelling, Residence in the Country, &c.: they offer nothing that deserves to be extracted.

A long chapter is devoted to the recommendation of Personal Cleanliness, a point certainly too little attended to in this country, where the mass of the population, indeed, have but few means of employing bathing as it ought to be used. There can be no doubt that very many of the almost infinite forms of diseases of the skin may be attributed to neglect on this point. The metropolis, indeed, is sadly deficient in establishments for bathing, both public and private; and, when we consider how easily water may be procured, we are astonished that the luxury of a bath is not to be met with in every private family.

M. Hufeland appears to be no friend to wearing woollen clothing next to the skin, though he notices some conditions of the system in which it ought to be preferred to linen.

The twelfth chapter treats of Diet and Temperance. M. Hufeland does not materially differ upon this very interesting and much-agitated subject, from the common opinions entertained by medical writers in this country. He approves of resting after meals; he praises water as a drink; and holds that a certain quantity of liquid is necessary with the meals. He recommends the mouth to be washed with cold water after each meal. He does not altogether reject beer or wine; but he says, and we think truly, that the latter, especially, contributes nothing to the prolongation of life. In conclusion, he is nearly as great an enemy to tobacco, in all its forms, as our worthy correspondent, Dr. KINGLAKE.*

A few pages are next devoted to Temper and the Disposition of the Mind, &c., the importance of which to health and long life no one will deny; but they are most commonly gifts of nature, and their acquirement is very difficult.

We must now hasten to the conclusion of this article.—There are many subjects still remaining to be treated in the last four chapters, but we do not think that they are of sufficient importance to detain our readers from more important matter: for example, the art of preventing and treating diseases never can be made of much general utility; nor are there any directions in the chapter relating to dangers from violent death, but such as are familiar to all professional men. A page or two of remarks upon Old Age, and the care which it requires, and a short chapter upon the Cultivation of the Physical and Moral Faculties, conclude the volume.

---

* See Dr. KINGLAKE's paper in our Number for December.

